# Beyond Common Ground: Dialogical Responsibility in Sustainable Human Resource Management

**DOI:** 10.1007/s10551-025-06086-7

**Published:** 2025-07-31

**Authors:** Jan Willem Nuis, Pascale Peters, Rob Blomme, Henk Kievit

**Affiliations:** 1 Nyenrode Business Universiteit, Breukelen, The Netherlands; 2 Ede Christian University of Applied Sciences (CHE), Ede, The Netherlands; 3 Innland University of Applied Sciences, Rena, Norway; 4 Open Universiteit, Heerlen, The Netherlands

**Keywords:** Pluralism, Dialogue, Dialogical responsibility, Frames, Sustainable HRM

## Abstract

Sustainable human resource management (HRM) advocates a shift toward pluralism, emphasizing the legitimacy of multiple stakeholder interests, voices, and meanings. Intended dialogue is often promoted as a key practice to support this shift. However, how intended dialogue actually contributes to pluralism remains under-theorized. Moreover, dialogue is frequently idealized as a route to harmony or ‘common ground,’ which can obscure power dynamics, silence dissent, and suppress difference. Building on dialogical ethics and framing theory, this conceptual paper introduces the *intended dialogical responsibility (IDR)* model, which reconceptualizes intended dialogue not as consensus-seeking but as a reflective space for exploring irreducible differences. Drawing on dialogue theorists such as Buber, Levinas, and Bakhtin, dialogical responsibility is proposed as an ethical form of ‘thirdness’—a mediating presence in the leader–worker dyad that invites participants to remain open to the other, question their own frames, and attend to asymmetries in voice and power. To support this stance, the IDR model employs framing theory to distinguish between micro-level situated frames (e.g., issue and relationship frames), meso-level strategic frames, and macro-level institutional frames. Dialogue is thus understood as a dynamic, ongoing, and reflexive process—occurring within and beyond episodic conversations—where meanings are co-constructed and contested over time. This paper contributes to sustainable HRM literature by offering a multilevel theoretical model and sensitizing questions to support ethically responsive dialogue. It provides a conceptual foundation for navigating pluralism and fostering dialogical spaces in complex organizational contexts.

## Introduction

In the last decade, sustainable Human Resource Management (HRM) has emerged as a normative paradigm criticizing the ethicality of mainstream HRM theory and practice characterized by ‘classic’ autocratic or managerialist/elitist unitarism (cf. Bray et al., 2020; Greenwood & Van Buren, 2017). In line with critical management literature (Bal & Brookes, 2022; Dundon & Rafferty, 2018; Janssens & Steyaert, 2009), sustainable HRM scholars have argued that HRM is overly skewed toward employers’ interests and, consequently, neglects individuals’ interests, power imbalances in employment relationships, and ignores the broader societal impacts of HRM (Ehnert et al., 2014; Greenwood, 2013; Kramar, 2014; Mariappanadar, 2012; Van Buren, 2020).

Given this, sustainable HRM scholars advocated a shift towards pluralism (Van Buren, 2020), presupposing that all parties in an employment relationship have different legitimate interests that cannot be dissolved into overarching, all-encompassing frames (Budd et al., 2021). More specifically, sustainable HRM has argued that HRM must be rooted in ethical values, such as responsibility, fairness, care, respect, and openness, to safeguard sustainability at the organizational, human, and societal levels (De Prins et al., 2014; Kramar, 2022). This implies that HRM must consider long-term human, social, and ecological interests and responsibly balance these with organizational interests (Ehnert et al., 2014; Kramar, 2014).

The shift towards pluralism elucidates three key ethical issues. First, one can no longer generalize ‘the employee,’ assuming identical or archetypal characteristics or interests (Benschop, 2001; Gilbert et al., 2015). Instead, since pluralism assumes that people are inherently different, their interests are never fully shared, prompting a shift in HRM policies and practices from sameness to difference. Second, challenging the traditional dominance of the employer’s voice, particularly within managerialist/elitist unitarism (Bray et al., 2020), underscores that, despite this prevailing one-sidedness, individuals embody a multitude of voices, expectations, and perspectives regarding the meanings of work, which coalesce within the workplace (Paulet et al., 2021). Third, by emphasizing the diversity of stakeholder backgrounds that pluralism presupposes, along with the complexity inherent in human meaning-making (Mowles, 2022), the assumption is challenged that clear, one-way communication between employers and employees is adequate to prevent ambiguity.

In light of this, sustainable HRM scholars have proposed dialogue at the workplace level as an intended practice that enables stakeholders (e.g., HRM practitioners, managers, and workers) to bridge differences in employment relationships (Ackers, 2019; Arenas et al., 2017; De Prins et al., 2014; Peters & Lam, 2015; Stankevičiūtė & Savanevičienė, 2018). This ‘intended dialogue’ refers to a mutually purposeful conversation in the leader-worker dyad intended to identify common ground and develop novel perspectives in employment relationships by encouraging authentic speaking, active listening, and mutual respect among participants (Nuis et al., 2021).

However, till now, studies that theorize and examine intended dialogue in the context of sustainable HRM are scarce. Moreover, the expected outcome of dialogue in facilitating the transition toward pluralism is ambiguous, lending it the status of a ‘silver bullet’ (Nuis et al., 2021; Peters et al., 2017). This echoes the concerns raised by Deetz and Simpson (2004, p. 141) that, reflecting on the dominant, liberal-humanistic dialogical perspective, its application often “foregrounds specific normative hopes” of finding common ground as an outcome by emphasizing the intrinsic value of individuals, mutual respect, and authentic communication. Still, it ignores how organizational and societal norms and existing power dynamics factor into its application, and presupposes a degree of feasibility and instrumentality. Therefore, combining positive expectations (e.g., expectations of enhanced equality, power symmetry, mutuality, voice) with vague notions of dialogue raises the risk of disappointment in employment relationships that may impede sustainable HRM’s intentions, as utilizing the term ‘dialogue’ does not render employment relationships pluralist by itself. Therefore, dialogue should not be idealized as inherently leading to harmony (Blok, 2014). Instead, it must be understood as a ‘space between’ people where differences—especially in interests, views, and power—emerge, and are recognized and explored (Janssens & Steyaert, 1999; Reitz, 2015).

This conceptual paper proposes cognitive framing theory (Hahn et al., 2014) as a lens that enables recognizing and exploring differences by focusing on people’s frames and framing processes in intended dialogues. First, this theory suggests that people organize and reduce perceptual information from societal, organizational, and micro-levels by linking it to existing cognitive frames they have developed over time and stored in their memory. A cognitive frame can be seen as a representation that wittingly and unwittingly guides people’s perceptions, interpretations, and inferences (Cornelissen & Werner, 2014). Frames not only shape how people interpret reality but also how they understand their voice in employment relationships (Budd & Bhave, 2008). Second, besides static notions of frames, framing theory elucidates the framing processes between people when engaging in intended dialogue in which framings may emerge (Dewulf et al., 2009). Intended dialogues can uncover framing differences, creating space for recognizing and exploring these differences along with the tensions they create.

Furthermore, this paper introduces the ethical imperative of ‘dialogical responsibility’ (Gardiner, 1996; Nealon, 1997) as a ‘third element’ (Janssens & Steyaert, 1999) in the leader-worker dyad to foster recognition and exploration of differences, making people aware of the ethicality of their frames and framing. Rather than attempting to reduce others to superficial or imposed sameness, it emphasizes the collective responsibility to intentionally explore both one’s frames and the other’s otherness. This signifies, reflecting sustainable HRM’s values, the commitment to being responsive to others and experiencing their otherness not as a threat but as an invitation to exploration. It entails recognizing and tolerating differences while embracing the understanding that ‘the other’ will remain unknowable (Gardiner, 1996).

This paper aims to contribute to sustainable HRM literature, specifically the position of intended dialogue, by developing a conceptual dialogue model, the ‘intended dialogical responsibility’ (IDR) model. Herewith, the following contributions are made.

First, recognizing people’s diverse interests, our dialogue model conceptualizes intended dialogue as a practice where differences between people can be recognized and explored without hastily aiming for ‘common ground’ as an outcome. This counters the ethical risk of fading difference in favor of superficial harmony (Blok, 2014). Second, drawing primarily from cognitive framing theory described by Cornelissen and Werner (2014), our dialogue model focuses on how cognitive frames at the micro-level shape micro-level interactions, and shape and are shaped by meso- and macro-level frames. This suggests various frames can be discerned in conversations representing different voices, inviting responsiveness and responsible dialogical exploration of differences. Third, our model incorporates interactional aspects of human relating, including framing processes that emerge in intended dialogue (Dewulf et al., 2009) and the influence of continuous local peer interactions outside intended dialogue. This provides a richer understanding of the ambiguities of human relating (Hahn et al., 2014; Mowles, 2022). Fourth, to facilitate dialogical responsibility in intended dialogue, our model theorizes a ‘dialogical space,’ the relational space between participants in which people explore framing differences as whole persons, not as roles or characterizations (Dewulf & Bouwen, 2012; Janssens & Steyaert, 1999; Marková, 2008). Finally, the dialogue model contributes to scholarly research and management practice by offering sensitizing questions that draw attention to the different frames that may emerge (Gillespie & Cornish, 2014).

## Dialogical Responsibility in Sustainable HRM

Reflecting insights from dialogue theorists Bakhtin (2011), Buber (1970), and Levinas (Arnett, 2012), the undisputed and often unnoticed assumption is that people can know what another person is, can be, or can become. Thereby, people ‘finalize’ others to understandable, handleable, and meaningful proportions that do not necessarily reflect the reality of the other. Following Bakhtin, ‘finalization,’ assuming to have a comprehensive view of the other through simplified reductions (frames like ‘the employee,’ ‘the employer,’ or ‘underperformer’), excludes the other ‘s otherness and reduces persons to the frames and framings that people have constructed of them (Benschop, 2001; Frank, 2005). Additionally, following Buber (1970), this process ignores the innate relational and communal inclination of people as it rationalizes, simplifies, and objectifies relationships between people from a subject-subject (I-You) to a subject-object (I-It) relationship (e.g., treating employees as tools for productivity without contemplating other needs and interests). Although the I-It relationship is a common way of human relating and not necessarily unethical, it becomes problematic when it is the only way of relating, risking alienating and depersonalizing people (Leicht-Deobald et al., 2021). Moreover, I-It relationships may become instrumentalized, and others are primarily valued for their “use-value” as a means to an end (Gardiner, 1996, p. 125).

In addition, Levinas argues that in face-to-face encounters, the other, in its vulnerability and otherness, demands one’s responsibility to take care of another human being without reliance on codified ethical rules or regulations, but from self-regulation: “In my face-to-face encounter with another person, I experience the primacy of the other who appeals to me and asks to act and behave ethically. This other is not an object of knowledge but a radical other who is unknowable, and simultaneously calls for ethical behavior in response to his or her call” (Blok, 2017, p. 2).

From a dialogical perspective, the difference between self and others is not resolved by pursuing shared or imposed identifications (e.g., job descriptions) or sublimated into a common ground at a higher level that everyone can agree with (e.g., explicit organizational values), but must be valued as a *conditio sine qua non.* Therefore, it can be argued that the proximity of the other as a human being necessitates openness and responsiveness from the ‘I’ to the ‘other’ (Gardiner, 1996) (the ‘You’ in Buber’s words) and acknowledgment of their inherent human dignity. For instance, an employee frequently misses work and demonstrates declining performance. The manager initiates a face-to-face dialogue to explore potential underlying factors, avoiding premature assumptions such as categorizing the employee as 'unmotivated' or initiating formal HR procedures like issuing a warning. During this dialogue, the supervisor uncovers that the primary cause of these issues is burnout due to a disproportionate workload which informs their mutual subsequent actions.

Therefore, people have a ‘dialogical responsibility’ to be responsive and to continuously question their finalized knowledge (e.g., frames) by intentionally exploring their implicit assumptions about others and their otherness in the workplace while acknowledging that ‘the other’ will never be fully understood (Arnett, 2012; Blok, 2014; Gardiner, 1996; McGhee, 2023; Murray, 2000; Nealon, 1997). In responsible dialogic workplace interactions, individuals commit to being aware of the generalizations, categorizations, and definitions in use (Bevan & Corvellec, 2007), accepting differences, tolerating tensions, exploring the origins of these tensions, and co-creating active responses that accommodate the multiplicity of perspectives. Conversely, dialogical irresponsibility is not genuinely seeking others to be authentically distinct as an ‘other’ but often a reassuring confirmation of one’s own identity through stereotyping, and reducing them to simplified sameness (Hammond et al., 2003).

Dialogical responsibility emphasizes the unique, intricate realities of individual local experiences and interactions over applying comprehensive, overarching ethical principles (Nealon, 1997). This becomes pivotal in giving substance to the values of sustainable HRM, such as respect, openness, and continuity of the operations of all people involved (De Prins et al., 2014). When people interact in intended dialogues, dialogical responsibility requires acknowledging inherent human differences as a value in itself, and taking a reflexive and exploratory approach to the other.

## Intended Dialogical Responsibility (IDR)

**Fig. 1:**
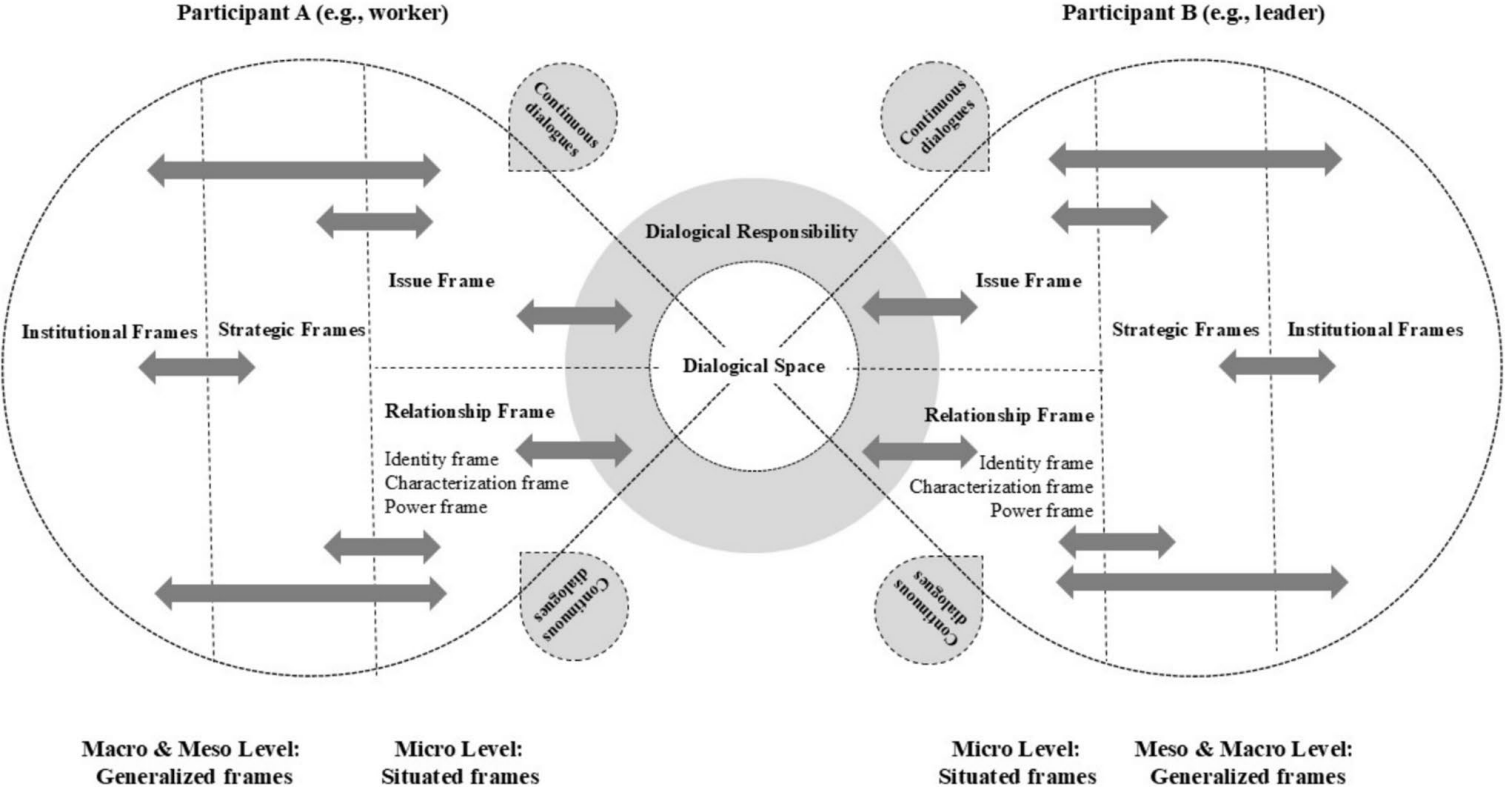
Intended dialogical responsibility model

This section will introduce the ‘Intended Dialogical Responsibity’ model (Fig. 1).

### Dialogical Exploration of Differences in Intended Dialogue

The shift to pluralism promoted by sustainable HRM redirects the focus of HRM policy and practice from sameness to highlighting differences. It critiques ‘classic’ unitarist frames of employment relations, which assume that what benefits the employer inherently benefits the employee (Budd et al., 2021). Moreover, unlike mainstream HRM, sustainable HRM does not treat employees as a homogeneous group but highlights their heterogeneity and distinct needs and interests (Van Buren, 2020). This implies that employee and organizational interests cannot be unified under a single viewpoint, as in managerialist/elitist unitarist HRM, but instead require a more differentiated approach. To address and bridge these differences, sustainable HRM literature advocates for intended dialogue (Ackers, 2019; Stankevičiūtė & Savanevičienė, 2018).

Intended dialogues are purposeful, conscious, and episodic conversations that foster responsible interaction, emphasizing participants’ attentive listening, voicing, respecting, suspending judgment, consciously seeking multiple perspectives, and amplifying unheard voices. It focuses on mutual understanding rather than persuasion, prioritizing tolerance over dominance, and balancing advocacy of one’s interests with exploration of others, often marked by structured turn-taking (Isaacs, 2009; Senge, 2006).

However, while intended dialogue fosters conditions for meaningful conversations, its intended outcomes are ambiguous. The dominant, liberal-humanist perspective in the dialogue literature emphasizes ‘common ground’ as an intended outcome (Deetz & Simpson, 2004). Dialogue embodies an ideal of harmony and unity (Blok, 2014) that helps participants align themselves with a third element, a ‘greater whole’ (i.e., common ground), beyond their individual perspectives. This shifts the dynamic between participants to a *trialectic* conversation, where the ‘third element’ or ‘thirdness’ influences and encompasses the personal perspectives of the participants (Dewulf & Bouwen, 2012; Janssens & Steyaert, 1999).

Despite these ‘normative hopes’ (Deetz & Simpson, 2004), the assumption that intended dialogue fosters harmony is idealistic, potentially problematic, and inhibits the shift to pluralism that sustainable HRM proposes. As a ‘technique’ for achieving harmony, dialogue can suppress the differences that dialogue is meant to address, overlook dominant voices’ implicit influence, and misinterpret human relationships. First, pursuing common ground as a third element in workplace dialogue does not necessarily eliminate differences. They may still exist, but are sidelined by the intention to achieve harmony. Rather than fostering mutual recognition, the desire for consensus may pressure participants to conform, subtly discouraging dissent or complexity. This undermines dialogical responsibility, which requires openness to the other’s distinct voice and perspective. Blok (2017) argues that seeking sameness risks neutralizing otherness, reducing the other as someone to be understood into one’s own frames, rather than engaging in their difference. In this light, ‘common ground’, as a third element, may not reflect shared understanding, but a soft form of persuasion that privileges agreement over recognition.

Second, the ideal of harmony may obscure how power distributions and prevailing organizational and societal norms shape what counts as common ground and whose voice dominates in shaping it (Deetz & Simpson, 2004). This normative view of harmony can mask asymmetrical power relations, where dominant groups impose their values and definitions of consensus, often marginalizing dissenting voices or alternative perspectives. As a result, what appears to be an agreement may reflect employee silence, as dissonance is suppressed (Grill et al., 2015).

Third, the idea that dialogue should culminate in harmony oversimplifies the nature of human relating. Dialogue is often regarded to transcend instrumental, subject-object relationships—what Buber (1970) termed I-It—toward genuine I-You, subject-subject encounters. This representation, however, oversimplifies the I–You relation by viewing it as the achievement of mutual understanding and harmony, where individuals fully recognize one another as human beings. As Friedman (1996) argues, human relationships are inherently marked by tension, ambiguity, and partial understanding; meaning and misunderstanding are always entangled. The presence of otherness ensures that people never mean precisely the same thing, and the moment we believe we fully understand the other, we risk finalizing them, reducing their otherness to a fixed identity.

In contrast, intended dialogue can be conceptualized as engaging with, not eliminating, differences and tensions in a space where people examine their assumptions and remain open, responsive to the other in their irreducible otherness. This calls for a shift to the responsiveness of all participants: an openness to questioning oneself and being questioned by the other, as in “(…) my dialogue with the other, my own interests and my own value frames become questionable” (Blok, 2014, p. 182). In this sense, intended dialogue transcends mere procedural conversational techniques; it embodies an ethical stance grounded in dialogical responsibility. This encourages an intended process of dialogical inquiry, where dialogue techniques like listening, suspending, voicing, and respecting (Isaacs, 2009) support this ethical engagement. In our model, this stance can be furthered and sustained by awareness of the reductive, coercive, and absolutist nature of one’s and others’ cognitive frames and the influence of framing processes.

### Exploring One’s Own and Others’ Cognitive Frames and Framings

Workplaces are submerged in overwhelming amounts of information, surpassing individual processing capabilities. To experience coherence in their situated experience, people deal with this abundance by developing cognitive frames, simplified reductions about themselves, others, issues, relationships, *et cetera* (Dewulf et al., 2009). These cognitive frames assist people in structuring and making sense of incoming perceptual information by integrating it with existing cognitive representations stored in their memory, thus enabling attention and perception, interpretation, prediction, and inferences (Cornelissen & Werner, 2014). Consequently, people tend to notice and accept new information (‘cues’) coherent with their a priori cognitive frames, which are reflexively activated (i.e., ‘remembered’) (Weick, 1995), and guide what people (expect to) experience in future situations (Tannen, 1979).

However, cognitive frames pose several ethical considerations. First, although the reductive nature of a frame enables insight and agency, pursuing an objective meta-position from which these reductions can be made is elusive, necessitating implicit, subjective, exclusive choices with normative implications (Cilliers, 2011). This is an ethical concern, as cognitive frames simplify complexity to simplicity, and, in human relations, reduce otherness to sameness (Cilliers & Preiser, 2010).

Second, as some cognitive frames may be regarded as more salient than others, attention can be coerced toward specific outcomes (i.e., ‘primed’), influencing judgment processes (Wang, 2007). Hence, frames influence what people believe the external environment represents in their view and, therefore, can be viewed as inherently biased and coercive (Dewulf et al., 2009; Woermann & Cilliers, 2012). For example, the autocratic unitarist frame (Bray et al., 2020) presupposes the manager as the single source of power to align diverse interests under the all-encompassing umbrella of the organization (Gilbert et al., 2015). This is ethically problematic, as it reduces employees to contributors to organizational outcomes and treats labor as a commodity (Budd et al., 2021). Moreover, this frame accentuates the normalcy of the manager’s voice in deciding what suits both the organization and the employee to pursue. It justifies coercion and power imbalances to attain this (Järlström et al., 2018). Conversely, a consultative unitarist frame, for example, acknowledges employee voices but retains decision-making authority with the employer, and a collaborative pluralist frame fosters differences in micro-level intended dialogues, acknowledging diverse voices (Bray et al., 2020).

Third, frames are often highly robust and long-lasting because they are “taken-for-granted beliefs about what is and what should be” (Van Buren, 2020, p. 694). Therefore, there is a risk that people absolutize their frames and impose them as norms at the expense of dialogically exploring others’ frames. Consequently, people may be generalized to measurable objects, or power imbalances may be taken-for-granted (Blok, 2017; Grossen & Salazar Orvig, 2011). This, in turn, can lead to the erosion of others’ unique identities and the subsuming of their multifacetedness into abstract systems of generalized expectations, effectively reducing them to mere embodiments of their assigned roles, functions, or one-dimensional characteristics (Gardiner, 1996).

Dialogical responsibility requires awareness of individuals’ cognitive frames’ reductive, coercive, and absolutist influence. To further elucidate and expand upon this, our IDR model (Fig. 1) distinguishes several frames at various levels: at the micro-level, ‘situated frames.’ At the meso- and macro levels, ‘generalized frames.’ These frames will be presented and summarized using *sensitizing questions* (Gillespie & Cornish, 2014): reflective questions that guide attention and interpretation of what is being communicated (Table 1).

**Table 1:** Sensitizing questions in the IDR model

Frame	Micro-level (observed individual framing)	Meso-/macro-level (influence of generalized frames)
Issue	How does each person articulate the core issue, concern, or opportunity? What language or metaphors are used?	What organizational or societal discourses seem to shape how the issue is framed or prioritized?
Identity	How does each participant position themselves (e.g., as expert, novice, peer)? What role claims or enactments are visible?	How do institutional roles or cultural expectations inform these self-positionings (e.g., manager, worker, profession)?
Characterization	How do participants construct the identity of the other? How is the other person described, addressed, or treated? What labels, assumptions, or relational cues are present?	What dominant narratives or role stereotypes appear to inform how the other is viewed (e.g., “just HR,” “the decision-maker”)?
Power	How is power expressed, negotiated, or? Who initiates, interrupts, or frames the conversation?	What structural conditions (e.g., hierarchy, norms, policies) seem to shape power relations in the exchange?

#### Micro-level Situated Frames: Issue and Relationship Frames

When people enter an intended dialogue to discuss an HRM issue, each person may have an a priori cognitive frame regarding the content, risks, or goals associated with the topics of concern (e.g., the HR issue at stake) (Dewulf & Bouwen, 2012). An ‘issue frame’ foregrounds a particular point of view, making specific interpretations more acceptable whilst dismissing other issue interpretations (Cornelissen & Werner, 2014). For instance, a manager and an employee may have different frames regarding the issue of ‘work-life balance:’ how employees manage the demands of their job alongside personal and family responsibilities. A manager might frame the issue in terms of flexibility—emphasizing efficient scheduling—while an employee may frame it around well-being, focusing on time for caregiving.

How people frame a particular issue also foregrounds whether it is considered ethical. This can be recognized in intended dialogues when people appeal to moral or deontological principles (e.g., fairness, justice, or respect) or organizational, societal, or relational responsibilities, such as caring, improving, or preventing negative externalities (Butterfield et al., 2000; Chen et al., 2020; Kreps & Monin, 2011). The sensitizing question: How does each person articulate the core issue, concern, or opportunity?

In addition to issue frames, people also develop cognitive frames that help them understand (employment) relationships (Moore et al., 2011). Relationship frames comprise three interrelated perspectives: the identity frame, relating to themselves; the characterization frame, associated with the other; and the power frame. First, the ‘identity frame’ describes how people see themselves and what people expect of themselves in a relationship as being part of a peer group (Bos et al., 2020). Developing an identity frame aims to uphold consistency or stability in social experiences (Dewulf et al., 2009; Moore et al., 2011). In some cases, people may frame themselves, for example, as victims in an employment relationship, while others may perceive themselves as valuable members of an organization. Identity frames are ethically laden because, in a relational sense, people always position themselves relative to others. Here, others are not only people they identify with in a positive sense (‘We’) but also others they frame in a negative sense and therefore exclude (‘Not-We’). An identity frame encompasses the nature of my identity and positionality within the context of this interaction with the other. Thus, the identity frame in employment relationships answers the sensitizing question: How do participants position themselves?

Second, in a relationship, the identity of the other is also framed: ‘characterization frames.’ This frame underlines positive, negative, or neutral reductive expectations of the other’s role, behavior, and identity (Mercuri, 2023). Characterization frames convey unquestioned, normalized reductions of the other that may invoke stereotyping. For example, some may frame managers a priori as ruthless pursuers of management interests, which guides subsequent expectations regarding managerial behavior at the workplace. A characterization frame involves reductive expectations of the other’s role, behavior, and identity within a relationship: How do participants construct the identity of the other?

Third, related to the previous two relationship frames, the ‘power frame’ refers to how people cognitively frame each other’s relative status to manage relationships, either being superior or inferior; who is expected to have formal or informal power, and how and by whom social choices are made. Hence, power frames lead people to perceive themselves as either more or less powerful compared to others (Shmueli et al., 2006). Power frames in employment relationships are a priori shaped by hierarchical position and formal authority. However, the perceived balance in people’s power frames can change as people become aware of their implicit need-based interdependencies between people that originate from others possessing or accessing valuable resources, such as time, wealth, knowledge, or authority (Avelino, 2021; Hammond et al., 2003; Leirvik, 2005). Thus, a power frame answers the question: How is power expressed, negotiated, or challenged?

#### Meso- and Macro-level Generalized Frames

The cognitive frames discussed so far were situated at the micro-level and based on people’s individual and workplace-level experiences. However, these never come about in a social vacuum but shape and are shaped by so-called ‘generalized frames,’ referring to organizational and societal expectations, values, and norms that influence people’s behavior on micro-levels (Cornelissen & Werner, 2014). More specifically, as social beings, people anticipate how their micro-level actions are viewed in their communities. In interactions, people anticipate how and how the other will respond, as well as the response of the ‘superaddressee: broader social, professional, organizational, and often unquestioned normative expectations (Barge & Little, 2002). This shows that “any discourse (even a dialogue with oneself) echoes the voices of discourses that were held elsewhere at other times and in other situations” (Grossen & Salazar Orvig, 2011, p. 492).

Therefore, generalized frames denote expectations that a dominant faction (e.g., management team, works council), a particular societal group, or a society at large has about employment relationships. Generalized frames and their associated norms and values are deeply ingrained in organizations, labor markets, and societies, along with their accompanying institutions, such as labor laws, professional discourses, and often governed by prevailing societal power distributions (Budd & Bhave, 2008; Geare et al., 2006; Greenwood & Van Buren, 2017).

Incorporating generalized frames into our conceptual model is crucial as it deepens our understanding of how frames, through socialization processes, are influenced by broader discourses, signifying generalized, taken-for-granted expectations about employment relationships that permeate people’s situated frames and ‘prescribe’ normalcy to people. Therefore, not only does the reductive, coercive, and absolutist nature of situated frames need inquiry, but also the influence of frames on other levels to foster dialogical responsibility. The general sensitizing question is: What broader norms, roles, structures, or expectations can be discerned in how participants see and handle the issue or their relationship?

Meso-level *strategic* frames refer to understanding a dominant group, often management, regarding how to make sense of what an organization is and what is essential (Kaplan, 2008). To garner support for a specific strategic direction or mitigate resistance against it, strategic frames deliberately use rhetorical devices that make a particular view more expedient and salient. Since management strategically employs purposeful communication to foster sensemaking, strategic frames often seem logical and inevitable, supported by power distributions. Given this, it is clear that organizations’ strategic frames can strongly impact micro-level relationships and issues as they ‘prescribe’ (give sense) what to value and pursue (Gioia & Chittipeddi, 1991).

On the macro-level, *institutional* frames describe the taken-for-granted normalities (societal expectations, values, and norms) that order and organize social and cultural experiences (Cornelissen & Werner, 2014). Following Goffman (1974), these shared cultural frames define and label experiences in specific micro-contexts, as through these frames, meaning can be imparted into experiences that, in turn, enable interpretation on a societal scale. Latent institutional frames can order and stabilize existing power relations and prime people to elaborate on customary roles and behavioral patterns associated with that specific frame (Cornelissen & Werner, 2014; Glaser et al., 2016). For example, in employment relations, both unitarist and pluralist frames are examples of macro-level frames that influence micro-level interactions, based on societal or professional discourses regarding work with performative influence on the meso-level and the micro-level, and vice versa (Bray et al., 2020; Sambrook, 2021).

#### Multilevel Interplay

The various frames in the model may appear independent, static, and hierarchical. However, it is essential to recognize that individuals’ cognitive frames are nested within generalized frames, which mutually shape each other. From a critical-realist perspective, the interplay between generalized and situated frames can be understood as the dynamic relationship between structure and agency (Delbridge & Edwards, 2013). Generalized frames, such as unitarist or pluralist frames, act as enduring structures that condition the actions and interpretations of individuals at the micro-level by embedding shared values and norms within organizational and societal contexts. These frames operate as enabling and constraining forces: they facilitate agency by offering salient understandings, concepts, or words for sense- and decision-making, while constraining it by limiting the range of acceptable interpretations or behaviors. For instance, in an HRM practice, a generalized frame like the expectation of ‘productivity’ permeates a manager-worker dialogue and guides managerial perception and action. The manager might emphasize performance metrics and adherence to industry standards, reflecting this broader norm. Conversely, the worker may prioritize job satisfaction as a precondition for productivity. In such interactions, generalized frames are shaped or renegotiated as they are taken up in micro-level conversations. However, the interaction also provides opportunities for reframing generalized frames. The worker’s reflexive responses, such as advocating for better work conditions to sustain performance, may challenge the macro-level frame and propose alternative practices. Over time, these micro-level reframings can influence meso-level dynamics, such as shifts in organizational policies, and may even ripple outward to reshape macro-level frames (Cornelissen & Werner, 2014). This highlights the reciprocal and dynamic nature of macro- and micro-level frames, where the reproduction of existing structures can lead to their eventual transformation. Dialogical responsibility, therefore, extends beyond the direct interaction in intended dialogues and comprises the interplay between generalized and situated frames. It requires moving beyond static notions of frames and engaging in how broader norms shape individual frames through reflexive and dialogic engagement.

### Exploring Framing Processes, Emergence, and Ambiguity

In intended dialogues, people’s cognitive frames do not simply ‘meet.’ As this represents an overly static view of human interaction, focusing on what happens “between the noses” (Dewulf et al., 2009, p. 162) is essential. In interactional ‘framing processes’ (Hahn et al., 2014), *framings* are produced and reproduced. When encountering framing differences, people may align their frames (frame alignment), co-construct new meanings that can alter a priori frames (i.e., reframing) (Cornelissen & Werner, 2014), or transcend these and collectively develop novel frames (Goffman, 1974). Moreover, people may negotiate meaning to resolve discrepancies, or engage in referencing and congruence to establish shared understandings (Dewulf et al., 2009). For instance, a worker raises concerns about a new scheduling policy, framing it as threatening trust and team morale. The worker’s issue frame values autonomy and well-being; relationally, she sees herself as a peer voicing collective concerns. The leader enters with a frame focused on efficiency and responsibility, carrying an identity frame as a decision-maker under pressure. Initial tension arises—one feels dismissed, while the other feels challenged. However, as the conversation unfolds, upholding dialogical responsibility, both adjust: the worker recognizes the constraints the leader faces, and the leader acknowledges the worker’s concerns as care, not resistance. They do not agree on the policy, but they gain insight into the different frames shaping their views—and into each other. These interactional processes continuously shape and reshape interaction and frames, sometimes reinforcing established frames, other times enabling emergent shifts.

This highlights another perspective beyond the time-limited intended dialogues: continuous dialogues (Nuis et al., 2021). As people usually spend a limited amount of time in intended dialogues (if any) in the workplace, most human interaction occurs in impromptu and informal workplace encounters (Barge & Little, 2002), which also shape and are shaped by frames (on various levels). Given the complex nature of human interaction and sensemaking in workplaces, people, collectively or individually, reduce and negotiate uncertainties of experienced social complexity and frame their work situations in continuous dialogical interactions (Cilliers & Preiser, 2010; Mowles, 2022; Weick, 1995). Therefore, the influence of informal conversations outside the intended dialogues must be considered in our model.

In this view, dialogue is fundamental to human consciousness, relationships, and the self, viewing people as inherently dialogic and dependent on others for sensemaking beyond episodes of intended dialogue. In this view, workplaces are complex microsystems consisting of diverse populations of people who share a common history of handling their bounded rationality (Kira & Lifvergren, 2014). Over time, in these dialogical interactions, emerging conversational patterns (e.g., recurring themes) may solidify into frames that influence how and what is discussed in microsystems and how experiences are valued to experience coherence. This implies that every intended dialogue in a dialogical space always takes place against a retrospective backdrop of reproduced history solidified in a priori frames of ongoing and historical interaction and collective meaning-making (Hahn et al., 2014; Maitlis & Christianson, 2014; Weick, 1995). As every participant in an intended dialogue is a member of multiple microsystems and, hence, is involved in various continuous dialogues, it is difficult to understand *where* frames originate and how another person should interpret this, which underlines the need to explore this ambiguity instead of adhering to the assumed unambiguousness of mainstream HRM.

Framing and continuous dialogues demonstrate that human relating always carries a sense of ambiguity, as new meanings can emerge. This challenges the unitarist assumption that employer interests are ‘unambiguously’ understood and accepted by all parties when clearly communicated, as these processes in microsystems complicate transferring one party’s intentions (e.g., management, HRM) to another. Instead, these intentions are constantly (re)constructed in ‘the living present’ (Mowles, 2022), emphasizing the interactional nature of dialogue at the heart of our conceptual model.

Therefore, dialogical responsibility encompasses and exceeds intended dialogues and emerging framings within them. Highlighting the continuous exploration of the ambiguities inherent in human interactions, prompting individuals to remain attentive, open, reflexive, and careful in conversations, honoring the diverse viewpoints that arise in both intended and continuous dialogues, and recognizing that finalized knowledge of other people does not exist. This signifies a dialogical perspective on organizational life and extends beyond merely facilitating dialogue (Friedman, 2001; Reitz, 2015).

### Dialogical Space: Dialogical Responsibility as Thirdness

Although physical conditions (e.g., seating of participants) and procedures influence the quality of intended dialogues (Isaacs, 2009), the dialogical space at the heart of the model, where ‘frames meet,’ is primarily the relational and reflective arena that emerges in ‘the space between’ people. It is an intersubjective space where meaning is not imposed, but where the diversity of voices is maintained without granting prior privilege to any perspective, suspending judgements, and enabling new meanings to emerge between those voices (Reitz, 2015).

The dialogical space is triadic: the influence of one on another is mediated by a ‘third element’ or ‘thirdness’. In the model, dialogical responsibility is positioned as a third element ‘outside’ the dialogical space, instead of common ground (Blok, 2014). This means that dialogical responsibility as a mediating element redefines the situation in which participants find themselves (Dewulf & Bouwen, 2012) and can shift the weight of the interaction beyond the dualities that framing differences evoke (Janssens & Steyaert, 1999). Framing becomes a shared inquiry, not a technique of influence. Framing contests (Kaplan, 2008) and misalignments (Dewulf et al., 2009) have not disappeared; instead, people recognize them. This enables people to become aware of the other’s otherness and acknowledge that one’s frames are reductions of reality with ethical implications (Woermann & Cilliers, 2012). Thirdness constitutes an ethical appeal made by the other—an invitation that participants must take seriously if they intend to engage in genuine recognition and exploration. Each person carries the quality of the encounter, and people need to engage one another with openness, presence, and willingness to be changed by the encounter, not as fixed roles or entities, but as mutually influencing participants (Anderson & Gehart, 2023; Reitz, 2015). Sustaining a dialogical space includes resisting the urge to dominate, close down, or prematurely resolve tensions, and instead holding space for the other without collapsing into agreement, avoidance, or authority (Bradbury & Lichtenstein, 2000).

A dialogical space is not a power-free space; rather, it is a power-aware space: people are aware of the role of positional authority, power framings, and generalized cultural norms that influence who gets to speak, whose frames are legitimized, and which meanings are considered valid (Grill et al., 2015). Leaders bear a heightened responsibility: their participation can open or foreclose dialogical space, which means becoming reflexive about how their framing shapes the interaction, making space for alternative voices, and being willing to relinquish control over outcomes. It requires exercising power-with, not power-over, and using authority to enable, not constrain, generative difference. If power remains unexamined, dialogue risks becoming performative, where differences are tolerated only within acceptable boundaries, and apparent openness conceals subtle forms of control (Bouton et al., 2024).

In practice, this means that people—especially those in positions of authority—commit to approaching one another dialogically, sustaining this mode even when the conversation becomes difficult and the differences between their frames seem irreconcilable. In those moments, dialogical responsibility truly counts—not as a technique, but as an ethical stance that opens the possibility of mutual transformation.

Figure 1 illustrates our multilevel conceptual model. It depicts two participants, Participant A (e.g., the worker) and Participant B (e.g., manager or leader), who engage in intended dialogue. Synthesizing insights from the previous sections allows for the development of a comprehensive analytical model using the sensitizing questions in Table 1.

## Discussion and Conclusion

The shift towards pluralism proposed by sustainable HRM required a more balanced view of the expected outcomes of intended dialogue between worker and leader. This paper contributes by proposing the multilevel ‘Intended Dialogical Responsibility model’ (IDR), which supports research and practice by enabling recognition and exploration of differences between people instead of striving for harmony as an intended outcome of dialogue (Blok, 2014).

To enable recognition and exploration of differences, our model applies framing theory as a lens to discern differences by focusing on the cognitive frames and framings in intended dialogues (Cornelissen & Werner, 2014). Moreover, how situated frames shape and are shaped by generalized frames that denote organizational and societal expectations (Budd et al., 2021; Kaplan, 2008). Furthermore, it raises attentiveness to continuous dialogue processes that influence situated and generalized frames, mainly through informal conversations outside the intended dialogues where individuals predominantly interact. It introduces dialogical responsibility as an ethical-laden third element (‘thirdness’) in the worker-leader dyad (Gardiner, 1996; Janssens & Steyaert, 1999). Dialogic responsibility requires participants to actively recognize and explore their frames regarding sustainable HRM issues such as well-being, work-life balance, career development (De Prins et al., 2014; Kramar, 2022), how they frame their relationship with others, and the effects of generalized frames on their views.

To discuss the usefulness of our model for scholarly research, the main points of the model and how this contributes to research will be discussed below, and questions for future research will be provided.

### Provisionality

Sustainable HRM presupposes the multiplicity of voices and calls for consciously examining our frames and interactions to identify and consider unheard voices, particularly those of employees (Paulet et al., 2021; Van Buren, 2020). Frames represent simplifications of complex, multifaceted, and dynamic realities at a particular point in time. Due to this, the epistemic status of a frame is provisional, as it is constantly revised in every interaction. Therefore, this impermanence of frames implies that people, over time, inevitably exclude perspectives when revising their frames. Temporality influences which voices are heard or silenced at specific points in time, depending on the context, prevailing norms, or evolving priorities. This ‘provisional imperative’ (Woermann & Cilliers, 2012), which addresses the ethicality of our frames from a temporal perspective, influences dialogical responsibility. The commitment to not finalize others must make people aware of the provisionality of our frames and cultivate the willingness and ability to consider alternative courses of action beyond the temporally constructed boundaries of current frames and recognize that these may evolve (Cilliers & Preiser, 2010). Research question: How does time influence perceptions of dialogical responsibility?

### Paradoxical Tensions and HR

While pluralism presents a meaningful, sustainable alternative frame for viewing the employment relationship, it also implies considering paradoxical tensions that become apparent when considering the employment relationship through frames other than the ‘classic’ unitarist (Bray et al., 2020). HR practitioners must simultaneously address the tensions that arise when balancing the diverse and legitimate interests of various stakeholders (Peters & Lam, 2015; Poon & Law, 2020). For example, Ehnert (2014) highlights the tension between efficiency (e.g., lean work processes), which enables financial continuity, and human sustainability (e.g., well-being), which allows sustainable work. Therefore, although pluralism opposes the ‘classic’ unitarist frame on ethical grounds, it simultaneously creates a new ethical problem: How to do justice to diverse interests and tensions?

However, the model does not explicitly address the role of HR practitioners, who are often tasked with designing HR practices that deal with these paradoxical tensions. However, line managers are responsible for implementation. Therefore, the development of intended dialogue, dialogical spaces, and dialogical responsibility requires dialogues between managers and HR professionals, who, in turn, also have divergent interests, views, and responsibilities (Bos-Nehles & Bondarouk, 2017). Research question: How does HR foster intended dialogue in contexts with multiple and paradoxical interests?

### Ambiguity and Complexity

As frames emerge in continuous dialogues in microsystems, multiple interpretations of issues and relationships may exist, contributing to the inherently ambiguous interpretation of work. Dialogical responsibility encourages participants to examine situated and generalized frames and focus on the dynamic processes that enable their emergence because these form the basis for paradoxical tensions. Continuous dialogues can lead to misunderstandings because differences in meaning often emerge due to the path dependence and complexity of human interactions. Following Cilliers, adding complexity and interactional thinking to our model "is not going to introduce us to a brave new world in which we will be able to control our destiny; it confronts us with the limits of human understanding" (Cilliers, 2002, p. 77). Moreover, as individuals are part of a web of relationships within and among microsystems, meaning becomes relational, retrospective, and non-representational, establishing an important caveat for our model (Lorino et al., 2011). This underscores that dialogical responsibility is a continuous, reflexive, and adaptive concept that embraces ambiguity and relationality as integral to navigating the complexities of meaning-making in organizational contexts.

The following research questions can be asked: What recovery activities occur if the continuous dialogues' impact causes misalignment in intended dialogues? How do participants in intended dialogues deal with speech confusion or generate novelty due to mismatched frames?

### Implications for Practice

First, dialogical responsibility, especially from leaders, requires *dialogic civility* (Arnett, 2001). It reminds leaders that their responsibility begins not with asserting truth, but with making space for the other in ethically responsive communication and to “keep the conversation going while examining differences” (Arnett, 2001, p. 325). It invites leaders to approach others with humility, respect, and attentiveness. For leaders, this means recognizing their embedded agency and exercising it with restraint, resisting control or self-centeredness, instead fostering conditions in which the voice of the other can emerge, even across power differences. Dialogic civility is essential for maintaining a dialogical space where both parties can engage from their respective standpoints without erasing their differences. In this way, it supports *intended dialogue* not as a technique, but as praxis: historically situated, responsive, and open to learning. These notions are essential for leadership development programs and education, as dialogic civility and dialogical responsibility require training (Ghoshal, 2005; McGhee, 2023).

Second, HR professionals must develop an understanding of HRM from a dialogical perspective, recognizing that employment relationships are shaped by diverse and sometimes conflicting cognitive frames. This approach requires HR practitioners to move beyond unilateral decision-making and instead facilitate intended dialogue. By sustaining dialogical spaces, HR professionals can enable employees and managers to share their perspectives, identify underlying assumptions, and address tensions constructively (Isaacs, 2009). This capacity enhances the alignment of organizational goals with employee well-being and fosters ethical, pluralistic practices that prioritize fairness, respect, and inclusivity in workplace interactions.

Third, since people have differing or opposing frames regarding work-related issues, power plays a significant role in ‘framing contests’ (Kaplan, 2008). These contests are shaped by power imbalances, which enable the enforcement of a dominant frame that reflects the interests of those in positions of authority, potentially marginalizing alternative views and limiting the capacity for genuine engagement dialogue. Recognizing, guiding, and influencing these power dynamics becomes a critical responsibility for HR practitioners. They must ensure that intended dialogues provide an equitable platform for all voices, particularly those that might otherwise be overshadowed.

Fourth, examining frames and framing processes is essential for HR practitioners to understand why their initiatives are sometimes perceived differently than intended. Individual variations in frames mean that people often interpret workplace cues, such as HR initiatives aimed at fostering sustainability, in ways that diverge from the intended outcomes (Nuis & Peters, 2021). To address this, HR practitioners can integrate insights from frame analysis to uncover how issues and relationships are framed in everyday interactions (Goffman, 1974). For instance, they could analyze how employees describe their roles and relationships or identify frame alignments during team discussions. This reflexive practice illuminates how generalized frames shape perceptions. Research questions with which voices can be made visible through framing research: How are issues and relationship frames reflected in interactions between people? How do institutional and strategic frames influence situated frames in the workplace?

Fifth, generalized frames “provide people with understandings of normative behavior and repertoires of potential action that shape their individual preferences and interests” (Glaser et al., 2016, p. 36). These frames establish a normality, influencing what individuals and organizations expect when discussing employment relations (Cornelissen & Werner, 2014; Glaser et al., 2016). They reinforce dominant norms and practices, shaping perceptions and guiding behavior, which can obscure alternative viewpoints or marginalize dissent. HR practitioners are shaped by these frames while actively reinforcing or challenging them. This necessitates a critical view of their assumptions and practices that align with or diverge from generalized norms. Recognizing this dual role challenges HR practitioners to remain reflexive and responsive in fostering equitable and sustainable workplace practices.

Beyond generalized frames, this notion has ethical implications for HR professionals. In transitioning toward pluralist employment relationships, HR professionals must reflect on their dual responsibility: legitimizing organizational interests while balancing diverse interests. This requires them to critically examine the source and ethical basis of their knowledge and actions, ensuring their interventions address and respect the varied frames of all stakeholders. HR practitioners’ role must be open to scrutiny—not only from external stakeholders but also from within their professional community. By critically engaging with the frames they embody and enact, HR professionals can challenge entrenched assumptions, foster inclusivity, and contribute to the creation of more pluralistic and ethically sustainable HR practices.

In conclusion, fostering sustainable employment relationships requires leaders and HR practitioners to go beyond finding common ground, as dialogical responsibility demands openness to differences and resisting the urge to reduce others to familiar terms or seek premature agreement. As their interpretations shape workplace interactions, leaders must adopt a reflexive, dialogical stance—recognizing that true dialogue involves responsiveness to what remains unresolved. HRM, must embrace this dialogical stance and develop policies and practices that enable exploration of differences, and true sustainable HRM outcomes.

## Data Availability

Not applicable for this theoretical paper.
